# Removal of an encrusted ureteral stent by cutting the stent with a holmium laser using 4.5‐Fr semi‐rigid and flexible ureteroscopes

**DOI:** 10.1002/iju5.12194

**Published:** 2020-08-01

**Authors:** Satoshi Imai, Takaaki Inoue, Mototsugu Muramaki, Yuji Yamada, Masato Fujisawa

**Affiliations:** ^1^ Department of Urology Amagasaki General Medical Center Amagasaki Japan; ^2^ Department of Urology Hara Genitourinary Hospital Hyogo Japan; ^3^ Division of Urology Department of Surgery Related Kobe University Graduate School of Medicine Kobe, Hyogo Japan

**Keywords:** encrusted ureteral stent, flexible ureteroscope, retrograde approach

## Abstract

**Introduction:**

Ureteral stents (double‐J stents) are widely used in urology to prevent or relieve ureteral obstruction and have become an integral part of urological practice. We have often experienced cases in which a stent cannot be removed due to encrustation.

**Case presentation:**

We describe the case of a 54‐year‐old male, who presented with a severely encrusted ureteral stent, which had only been inserted for one month until second surgery for renal stones. The ureteral stent could not be removed as it had become encrusted with renal stones. The encrusted ureteral stent was successfully removed by cutting it with a Ho:YAG laser using 4.5/6.5‐Fr semi‐rigid and flexible ureteroscopes retrogradely. The patient subsequently remained stone‐free without any complication.

**Conclusion:**

We experienced a case in which an encrusted ureteral stent was successfully removed retrogradely. Technological advancements in endourology will hopefully make the treatment of such cases safer and less invasive.

**Abbreviations & Acronyms**
CT: computed tomography
ECIRS: endoscopic combined intrarenal surgery
ESWL: extracorporeal shock wave lithotripsy
KUB: kidney ureter bladder
PCNL: percutaneous nephrolithotomy
RIRS: retrograde intrarenal surgery
URL: ureteroscope lithotripsy
URS: ureteroscope

**Keynote message**

There are various management strategies for encrusted stents. Advances in medical equipment have resulted in the development of less invasive treatments. We successfully removed the stent by cutting it with a Ho:YAG laser using 4.5/6.5‐Fr semi‐rigid and flexible URS.

## Introduction

Many urologists have experienced cases in which an encrusted ureteral stent cannot be removed. The encrusted stent can have potentially lethal complications, such as recurrent urinary tract infections, hematuria, obstruction, and renal failure.^1^, ^2^

Previous studies have reported various management strategies for encrusted stents, such as ESWL, URL, PCNL, and open surgery.^3^, ^4^, ^5^, ^6^, ^7^, ^8^

Advances in medical equipment have resulted in the development of less invasive treatments. Recently, Thomas *et al*. reported a retrograde ureteroscopic approach performed with a Ho:YAG laser, which made it possible to remove almost all encrusted and retained ureteral stents.^9^

He *et al*. reported that they could reduce the need for PCNL by using a 4.5/6.5‐Fr semi‐rigid URS for retrieving retained encrusted ureteral stents.^10^ 4.5/6.5‐Fr semi‐rigid URS is made up of 4.5‐Fr tip and 6.5 shaft.

We report a case in which endoscopic treatment was successfully performed for treatment of encrusted ureteral stent via a retrograde approach using semi‐rigid and flexible URS.

## Case presentation

We present the case of a 54‐year‐old male, who underwent RIRS and ESWL several times for ureteral stones.

In July 2019, a staghorn calculus was detected on a CT scan. ECIRS was performed for the staghorn calculus. A ureteral stent was also inserted after the surgery. One month later, we decided to perform RIRS for the residual stones.

**Fig. 1:**
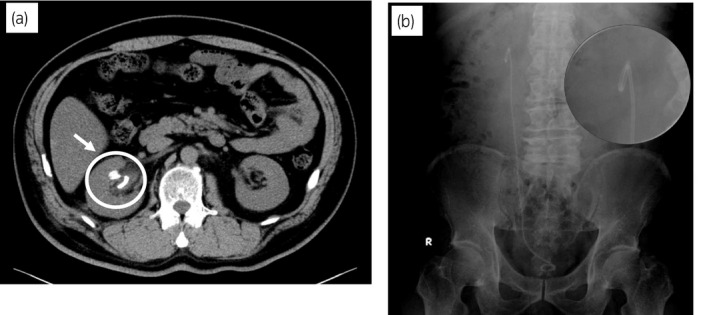
(a) Plain CT showing residual stone. (b) The preoperative imaging that X‐ray KUB did not show encrusted ureteral stent.

An imaging study including KUB and CT revealed a residual stone in the lower pole, measuring 0.8 × 0.7 cm, beside the ureteral stent without hydronephrosis (Fig. 1a). The preoperative imaging did not show that stones had formed on the stent (Fig. 1b).

The patient was administered prophylactic antibiotics before the operation, and was oriented in the lithotomy position under general anesthesia.

**Fig. 2:**
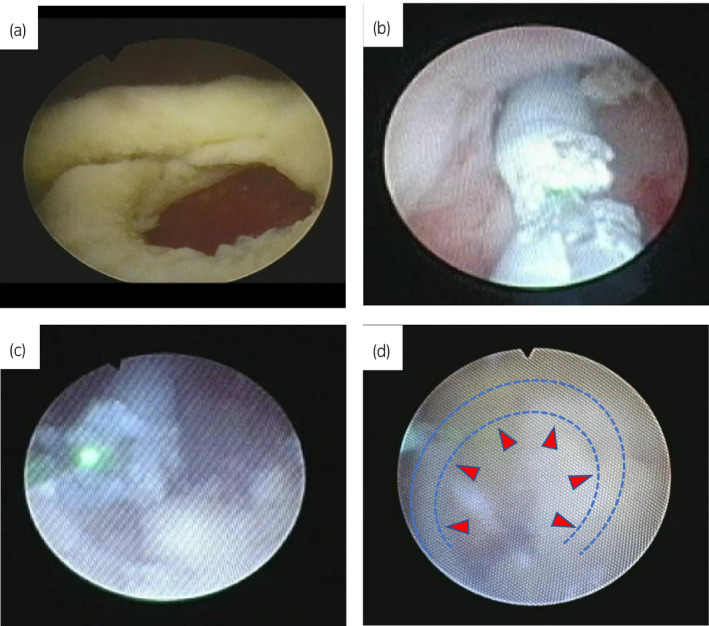
(a) Cystoscopic view showing the distal end of ureteral stent. (b) The stent was cut by the Ho:YAG laser by using 4.5‐Fr semi‐rigid URS. (c) The stent was cut by the Ho:YAG laser by using frexible URS. (d) The frexible URS view showing the proximal end of ureteral stent.

We noticed the distal end of the encrusted ureteral stent when we inserted a cystoscope into the bladder (Fig. 2a).

We could not pull out to outside external urethral meatus the encrusted stent by using forceps.

Therefore, we inserted a 0.035‐inch guidewire with a hydrophilic coating beside the retained ureteral stent and confirmed that the proximal side of the guidewire had been placed in the collecting system, under fluoroscopic guidance.

Then, we crushed the encrusted part of the distal end of the ureteral stent in the bladder using a laser, before creating a space beside the ureteral ostium, and inserted a 4.5/6.5‐Fr semi‐rigid URS.

4.5/6.5‐Fr semi‐rigid URS could be inserted up to U2 (L5 level), due to the resistance for ureteral stricture, and the stent was cut in the middle portion by using a laser on the 1.0 × 5 Hz setting (Fig. 2b).

However, the URS could not advance into the ureter due to the resistance for ureteral stricture (Fig. 2b).

Subsequently, we switched from a semi‐rigid URS to a flexible URS (Flex‐X2; Karl Storz, Culver City, CA, USA) after inserting a 10/12‐Fr 35 cm ureteral access sheath (Proxis™ Ureteral Access Sheath; BARD Medical, West Sussex,UK) into the ureter in front of the stricture portion.

Then, the flexible URS was advanced into the ureter through the ureteral access sheath.

After cutting the stent, the remaining encrusted upper curl was pushed back into the renal pelvis. We were able to remove all of the stent using basket forceps (N‐Circle Stone Extractor; Cook Medical, Bloomington, IN, USA) (Fig. 2c).

**Fig. 3:**
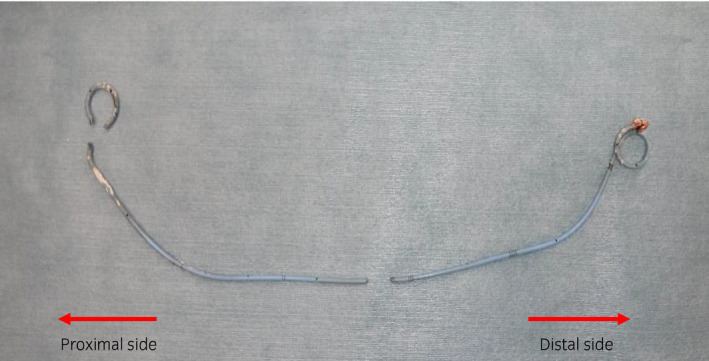
Ureteral stent separated and removed in three parts.

Surgical findings indicated that diffuse encrustations completely encasing both of the pigtail and ureteral portions (Fig. 3).

After that, the remaining stones were subjected to RIRS as usual and lithotripsy. The operation was completed without a postoperative drainage stent after confirming that there were no remaining stones. We avoided placement of a postoperative drainage stent to worsen the patient's quality of life.^11^ The operative time was 70 min, and the stones were composed of 97% calcium phosphate and 3% calcium oxalate.

The patient did not have any postoperative complications and was discharged on postoperative day 1.

## Discussion

In the absence of clear guidelines about the removal of retained stents, this problem has been managed with a variety of treatment modalities. These methods include various combinations of PCNL, ureteroscopy with laser lithotripsy, cystolitholapaxy, and ESWL.^1^

The rate of ureteral stent encrustation is dependent on the composition of the patient’s urine, the patient’s infection status, a history of urolithiasis, infections, and the duration of stenting are regarded as important risk factors for encrustation.^12^ However, it is unclear how long stent encrustation takes. In 2009, Acosta‐Miranda *et al*. classified covered ureteral stents and proposed a treatment algorithm. This classification is defined as grade I: minimal linear encrustations along either of the pigtail portions; grade II: circular encrustation completely encasing either of the pigtail portions; grade III: circular encrustation completely encasing either of the pigtail portions as well as linear encrustation of the ureteral aspects of the indwelling ureteral stent; grade IV: circular encrustations completely encasing both of the pigtail portions; and grade V: diffuse and bulky encrustations completely encasing both of the pigtail and ureteral portions.^13^ The present case is classified as grade IV.

According to the latter report, PCNL is recommended for cases in which stones form in the proximal curl of a stent.

However, PCNL requires two or three operators who are experienced in endoscopic surgery, and it also requires equipment, such as a monitor and a laser generator. For this reason, compared with endoscopic surgery much fewer facilities in Japan can perform PCNL. Conversely, our surgical method could be carried out at many facilities.

In 2019, Thomas *et al*. reported that both rigid and flexible URS were required to treat 46 (90%) of 51 encrusted stents.^9^ If the proximal tail of a stent is encrusted with stones, as was true in the present case, it might be difficult to treat with either type of URS alone. In addition, Thomas *et al*. used 12/14‐Fr ureteral access sheath in 37 cases (72%), but we used 10/12‐Fr to ensure that the ureter was treated gently. We consider that a thin sheath should be used whenever possible. Double‐J stents were inserted for postoperative drainage in 40 of the 51 patients (78%) in the latter study. The authors did not disclose the reason for placing the stent, but if the ureter is in good condition after surgery, the stent placement can be avoided as in this case.

## Conclusion

We experienced a case involving a grade IV encrusted ureteral stent, and successfully removed the stent by cutting it with a Ho:YAG laser using 4.5/6.5‐Fr semi‐rigid and flexible URS. Developments in endourological medical equipment make it possible to remove such stents via a retrograde approach alone.

## Conflict of interest

The authors declare no conflict of interest.
